# Mean platelet volume and its relationship with carotid atherosclerosis in subjects with non-alcoholic fatty liver disease

**DOI:** 10.3109/03009734.2010.500062

**Published:** 2010-10-27

**Authors:** Guldem Kilciler, Halil Genc, Serkan Tapan, Fatih Ors, Muammer Kara, Nuri Karadurmus, C. Nuri Ercin, Yildirim Karslioglu, Selim Kilic, Sait Bagci, M. Kemal Erbil, Teoman Dogru

**Affiliations:** ^1^Department of Gastroenterology, Gulhane School of Medicine, Etlik, AnkaraTurkey; ^2^Department of Biochemistry, Gulhane School of Medicine, Etlik, AnkaraTurkey; ^3^Department of Radiology, Gulhane School of Medicine, Etlik, AnkaraTurkey; ^4^Department of Internal Medicine, Gulhane School of Medicine, Etlik, AnkaraTurkey; ^5^Department of Pathology, Gulhane School of Medicine, Etlik, AnkaraTurkey; ^6^Department of Epidemiology, Gulhane School of Medicine, Etlik, AnkaraTurkey

**Keywords:** Fatty liver disease, mean platelet volume

## Abstract

**Background:**

Non-alcoholic fatty liver disease (NAFLD) is linked to an increased risk of cardiovascular disease. Mean platelet volume (MPV), a determinant of platelet activation, is an emerging risk factor for atherothrombosis.

**Aims:**

The aim of this study was to investigate the levels of MPV in subjects with NAFLD having no confounding factors for atherosclerosis such as obesity, diabetes mellitus, and hypertension. In addition, the possible relationship between MPV and carotid artery intima media thickness (CIMT), a well known marker of subclinical atherosclerosis, was also studied.

**Methods:**

MPV and CIMT levels were measured in 60 biopsy-proven NAFLD subjects and 54 healthy controls. Age and sex were similar between two groups.

**Results:**

Body mass index and waist circumference levels were higher in the NAFLD group when compared to the controls. There were no differences between the two groups regarding LDL cholesterol levels, whereas HDL cholesterol levels were lower in the NAFLD group. MPV and CIMT levels were not different between the two groups. According to the correlation analyses, CIMT levels were positively correlated to age in patients with NAFLD. However, no significant correlation was found between MPV and CIMT levels.

**Conclusions:**

The results of this study do not show any difference in MPV levels between subjects with NAFLD and controls. These finding suggests that in the absence of other metabolic risk factors, MPV might not be involved in the mechanism(s) of increased cardiovascular risk in NAFLD.

## Introduction

Non-alcoholic fatty liver disease (NAFLD) is increasingly diagnosed worldwide and considered to be the commonest liver disorder in clinical practice. It comprises a disease spectrum from variable degrees of simple steatosis (SS) to non-alcoholic steatohepatitis (NASH) and cirrhosis. SS is benign, whereas NASH is characterized by hepatocyte injury, inflammation, and fibrosis which can lead to cirrhosis, liver failure, and hepatocellular carcinoma (1,2).

NAFLD is strongly associated with insulin resistance, obesity, hypertension, and dyslipidemia and is now regarded as the liver manifestation of the metabolic syndrome (3). Subjects with NAFLD have a higher prevalence of atherosclerosis, as shown by increased carotid artery intima media thickness (CIMT), numbers of atherosclerotic plaques, and circulating markers of endothelial dysfunction (4–6). Thus, a number of studies have reported the increased prevalence of cardiovascular, cerebrovascular, and peripheral vascular diseases in subjects with NAFLD (7,8). Although an indirect association between NAFLD and cardiovascular disease (CVD) is reported, a growing body of evidence supports a direct role for NAFLD in the pathogenesis of atherosclerotic CVD (9).

Platelets, whose size mainly depends on the degree of fragmentation of megakaryocytes, are anucleate cells (10). It is known that large platelets have a greater content of granules and can therefore exert their haemostatic, vasomotor, and pro-inflammatory functions with greater efficacy, so increased platelet activation is associated with increased platelet volume (11,12). Platelet activation may also be associated with microvesicle formation (smaller platelets). Mean platelet volume (MPV), a determinant of platelet activation, is an emerging risk factor for atherothrombosis (13). The increase in MPV may take place in acute myocardial ischemia (14), acute myocardial infarction (15), coronary atherosclerosis (16), the presence and the short-term prognosis of stroke (17) and the long-term risk of stroke (18). Also, case-control studies have documented significant associations of MPV with type 2 diabetes mellitus (T2DM) (19), pre-diabetes (20), obesity (21), and other metabolic risk factors (22,23).

The biological mechanisms by which NAFLD might contribute to accelerated atherosclerosis are still poorly understood. The main aim of this case control study was to investigate the association of NAFLD with MPV levels as an independent risk factor of atherosclerosis. In addition, the possible relationship between MPV and CIMT, as well as being a known marker of subclinical atherosclerosis, was also investigated. In order to prevent any interference of confounding factors for inflammation or atherosclerosis, we studied a specifically selected group having no additional disorders such as hypertension, diabetes mellitus, or obesity.

## Methods

### Subjects

A total of 60 male subjects with biopsy-proven NAFLD referred to the out-patient clinic of the Department of Gastroenterology, Gulhane School of Medicine, Ankara, Turkey were enrolled in the study. Inclusion criteria were: persistently (for at least 6 months) elevated aminotransferases, ultrasonographic presence of hyperechogenic liver without any other liver or biliary tract disease, liver histology compatible with a diagnosis of NASH or SS. Exclusion criteria were: a history of alcohol consumption 40 g/wk, as assessed by a detailed interview extended to family members, obesity (BMI ≥30 kg/m^2^), hypertension, positive blood markers of viral, autoimmune, or celiac disease, abnormal copper metabolism or thyroid function tests, a diagnosis of overt diabetes mellitus (fasting blood glucose ≥7 mmol/L or ≥11.1 mmol/L at 2 hours on a standard oral glucose load, oral glucose tolerance test), total cholesterol (TC) ≥6.47 mmol/L, triglycerides (TG) ≥4.52 mmol/L, exposure to occupational hepatotoxins or drugs known to be steatogenic or to affect glucose and lipid metabolism. The control group consisted of 54 healthy male subjects, matched for age, with normal liver enzymes and abdominal ultrasonography. All participants provided a medical history and underwent a clinical examination. The weight and height of the participants were measured with a calibrated scale after the patients had removed their shoes and any heavy clothing. Body mass index (BMI) was computed as body weight/(height^2^). Waist (narrowest diameter between xiphoid process and iliac crest) and hip (widest diameter over greater trochanters) circumferences to derive waist-to-hip ratio (WHR) were measured as well.

The study was approved by the local ethics committee of Gulhane School of Medicine, and all participants signed informed consent.

### Biochemical measurements

For biochemical analyses, all blood samples were collected from an antecubital vein, between 08.00 and 09.00 a.m. after an overnight fasting. The samples were centrifuged for 15 minutes at 4000 rpm, aliquoted and immediately frozen at −80°C for analyses until assayed. All samples were run in the same batch. Fasting plasma glucose (FPG), TC, TG, and high-density lipoprotein cholesterol (HDL-C) levels were measured by the enzymatic colorimetric method with Olympus AU2700 autoanalyzer using reagents from Olympus Diagnostics, (GmbH, B). Low-density lipoprotein cholesterol (LDL-C) was calculated by Friedewald's formula (24).

The serum basal insulin level was measured in duplicate by the electrochemiluminescence method with Modular Analytics E170 autoanalyzer using reagents from Roche Diagnostics (Mannheim, Germany). Insulin resistance was calculated by modified homeostasis model assessment of insulin resistance (HOMA-IR), with the following formula: HOMA-IR = fasting plasma insulin (μU/mL) × fasting plasma glucose (mmol/L)/22.5. HOMA-IR was originally reported by Matthews et al. (25), and this index has been shown to be well correlated with the results of the euglycemic–hyperinsulinemic clamp method to determine insulin resistance (26).

MPV levels were measured in a blood sample collected in citrate (1:4 v/v) in order to avoid the platelet swelling induced by EDTA (27). An ABX Pentra 120 automatic hematology analyzer (Horiba ABX Diagnostics, Montpellier Cedex, France) was used for whole blood counts.

### Histopathologic analysis

Liver tissue was stained with hematoxylin-eosin, reticulin, and Gomori trichrome stains and scored by an experienced pathologist. All cases showed macrovesicular steatosis affecting at least 5% of hepatocytes and were classified as steatosis. In addition to steatosis, the minimum criteria for the diagnosis of steatohepatitis included the presence of lobular inflammation and either ballooning cells or perisinusoidal/pericellular fibrosis in zone 3 of the hepatic acinus. All cases were scored using the method of Brunt (28). Steatosis was graded as follows: grade 1 (5%–33% of hepatocytes affected); grade 2 (33%–66%); or grade 3 (>66% of hepatocytes affected). Fibrosis was assessed with the Mason trichrome stain. Other histological features evaluated in hematoxylin-eosin sections included lobulillar inflammation and portal inflammation.

### CIMT measurement

The assessment of the CIMT was performed by one experienced radiologist who was blinded to the diagnosis of participants. High-resolution B-mode ultrasound images were obtained with use of a 7.5-MHz transducer (Power Vision 8000, Toshiba Shiomoishigami, Otawara-Shi, Japan). The right and left common carotid artery and the carotid bifurcation-bulb areas were scanned from multiple planes. Images were obtained from the far wall of the distal 10 mm of left and right common carotid arteries at a site free from any discrete plaque.

### Statistical analysis

Results are reported as the mean ± SD and median (min–max). The Kolmogorov-Smirnov test was used to determine the distribution characteristics of variables, and Levene's test was used to evaluate the equality of variance. Differences between groups were tested for significance by independent samples *t* test and Mann-Whitney *U* test, as appropriate. The relationship between variables was analyzed by Spearman's rho correlation. Differences and correlations were considered significant at *P* < 0.05.

## Results

Table I shows the main characteristics and the laboratory data of the patients and the controls. Age was similar between the two groups. BMI and WC levels were higher in the NAFLD group when compared the controls.

**Table I.** The characteristics of the subjects with non-alcoholic fatty liver disease (NAFLD) and controls.

	NAFLD (*n* = 60)	Controls (*n* = 54)	*P*
Age (years)	31.7 ± 5.6	30.3 ± 5.6	0.17^a^
BMI (kg/m^2^)	26.7 ± 1.9	23.6 ± 2.1	<0.001^a^
WC (cm)	93.8 ± 4.3	86.5 ± 6.1	<0.01^a^
FPG (mmol/L)	5.06 ± 0.69	4.52 ± 0.47	<0.001^a^
TC (mmol/L)	5.27 ± 1.08	4.73 ± 0.79	<0.005^a^
TG (mmol/L)	1.91 (0.25–4.22)	1.22 (0.58–3.28)	<0.001^b^
HDL (mmol/L)	1.1 ± 0.18	1.25 ± 0.27	0.01^a^
LDL (mmol/L)	3.06 (0.62–5.69)	2.74 (1.55–4.42)	0.29^b^
ALT (IU/L)	89 (37–207)	16.5 (6–40)	<0.01^b^
AST (IU/L)	41 (20–148)	20 (11–35)	<0.01^b^
Insulin (μU/mL)	9.8 (2.1–31.3)	6.7 (3.1–18.5)	0.01^b^
HOMA-IR	2.18 (0.34–7.38)	1.46 (0.59–4.06)	<0.001^b^
MPV (fL)	8.9 ± 1.1	8.8 ± 0.9	0.77^a^
CIMT (mm)	0.59 ± 0.11	0.56 ± 0.06	0.23^a^
Histology:			
Fat score (1/2/3) (%)	60/28/12	-	-
Necro-inflammation score (0/1/2/3) (%)	10/72/18/0	-	-
Fibrosis stage (0/1/2/3/4) (%)	55/40/3/2/0	-	-

The data are presented as the mean ± SD or median (minimum–maximum).

^a^Independent sample *t* test.

^b^Mann-Whitney *U* test.

BMI = body mass index; WC = waist circumference; FPG = fasting plasma glucose; TC = total cholesterol; TG = triglyceride; HDL-C = high-density lipoprotein-cholesterol; LDL-C = low-density lipoprotein-cholesterol; ALT = alanine aminotransferase; AST = aspartate aminotransferase; HOMA-IR = homeostasis model assessment of insulin resistance; MPV = mean platelet volume; CIMT = carotid intima media thickness.

**Figure 1.**
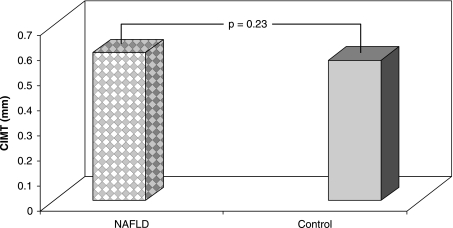
CIMT levels in subjects with NAFLD and controls. There were no differences between two groups according to CIMT levels.

**Figure 2.**
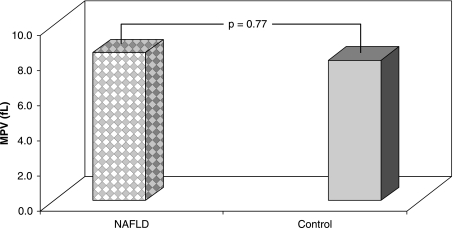
MPV levels in subjects with NAFLD and controls. There were no differences between two groups according to MPV levels.

MPV and CIMT levels were not different in the two groups (Figures 1 and 2). FPG, TC, and TG levels were higher in the NAFLD group than in the controls. There were no differences between the two groups regarding LDL-C, whereas HDL-C levels were lower in the NAFLD group (Table I).

According to the correlation analyses, CIMT levels were positively correlated to age in patients with NAFLD (*r* = 0.37, *P* = 0.03). However, no significant correlation was found between MPV and CIMT levels.

## Discussion

This preliminary study has shown that MPV levels are not different in subjects with NAFLD who have no confounding factors such as hypertension, diabetes, and obesity, in comparison with healthy controls. The abnormalities in glucose and lipid metabolism and also blood pressure that can potentially affect endothelial function are frequently accompanied by NAFLD. Therefore, including only subjects free from any confounding factor is an important feature of the present investigation.

Insulin resistance, oxidative stress and subsequent lipid peroxidation, pro-inflammatory cytokines, adipokines, and mitochondrial dysfunction are the main pathogenetic factors in the development of NAFLD. Furthermore, accumulating evidence supports an association between NAFLD and metabolic syndrome (2,3). Although the data are mainly epidemiological, NAFLD and metabolic syndrome seem to have common pathophysiological mechanisms with focus on insulin resistance as a key factor. On the other hand, the existence of cardiovascular involvement in NAFLD is well established. Accumulation of abdominal fat seems to be the key factor driving both fatty liver and carotid artery disease (29). NAFLD is a strong risk factor for increased carotid intima media thickness (30) and reduced endothelial function (31). However, whether NAFLD is a consequence of or a contributor to the dysmetabolic cascade that occurs in insulin resistance and metabolic syndrome is not well established. Experimental and human studies suggest that both mechanisms are probably involved. There is, as yet, no direct evidence that reducing liver fat is beneficial for cardiovascular morbidity and mortality, and a large majority of patients with NAFLD probably might die of cardiovascular disease before they develop liver failure. Overall, the current body of evidence strongly suggests that NAFLD is likely to be associated with increased CVD risk and raises the possibility that NAFLD may be not only a marker but also an early mediator of atherosclerosis (32).

Platelet volume is a marker of platelet function and activation. It is measured as MPV by clinical hematology analyzers (13). Larger platelets are more reactive than smaller ones and produce more prothrombotic factors. On the other hand, endothelial dysfunction favors platelet activation (33). In studies with T2DM patients and subjects with impaired glucose tolerance, MPV levels were found to be higher compared to controls, and it is proposed that higher MPV levels may play a role in the micro- and macrovascular complications related to T2DM (19). MPV levels in patients with obesity were found to be higher compared to controls, and there was a positive correlation between obesity and MPV levels (21). Besides, it was shown that weight loss in obese patients was associated with a reduction in MPV levels (34). In hypertensive patients, elevated MPV levels have also been reported (23). Hypercholesterolemia, another risk factor of atherosclerosis, has effects on platelet function such as platelet size (measured as MPV), platelet aggregation, and platelet activation (35). Consequently, in order to reduce the effect of metabolic factors, mentioned above, we designed this study with patients who have no confounding factors such as hypertension, diabetes, and obesity. We did not find a significant difference regarding the MPV between the two groups. In addition, there were no relations between MPV and CIMT levels and other metabolic parameters such as glucose and lipids. Overall, this finding suggests that in the absence of other metabolic risk factors, MPV per se is not involved in the mechanism(s) of increased cardiovascular risk in NAFLD.

CIMT is a reliable index of subclinical atherosclerosis, and epidemiologic studies have demonstrated that there is a significant association between CIMT and CVD (36,37). In a systematic review, including seven studies with 1427 patients and 2070 controls, increased CIMT levels were reported in NAFLD when compared to controls (38). When these studies were analyzed separately, some of the patients with NAFLD had metabolic confounders like obesity and T2DM (39–41). It has been reported that CIMT levels may be affected by these metabolic risk factors. So, we think that some of the previous reports regarding the CIMT in NAFLD might be affected by these metabolic confounders. In addition, the age of the study participants were older in the above-mentioned studies, and it is well known that there is a strong and independent relationship between age and CIMT. In the present study, we found no differences in CIMT levels between subjects with NAFLD and controls. However, there was a significant correlation between age and CIMT in subjects with NAFLD. In light of these findings, we suggest that NAFLD is not independently associated with carotid atherosclerosis in people who have no metabolic abnormalities, and age may be an important determinant for the development of atherosclerosis in NAFLD. Thus, our findings are consistent with the results of recently published reports about this issue (42–44).

There are two limitations of the present study. Firstly, through the sample size and the strict inclusion criteria, the findings obtained are not representative for all subjects with NAFLD. But we think that the design of our study was a requirement to achieve the goals. Secondly, though it is simple, non-invasive, and known to be correlated well with the clamp test, the HOMA formula used to calculate insulin sensitivity in this work is only an estimate and cannot be as accurate as the euglycemic–hyperinsulinemic clamp method.

In conclusion, the results of this preliminary report do not show any difference in MPV levels between subjects with NAFLD and controls. These findings suggest that in the absence of other metabolic risk factors MPV might not be involved in the mechanism(s) of increased cardiovascular risk in NAFLD.
